# Assessment of the underlying causes of adult deaths using a short version of verbal autopsy in Xaiyabouli Province, Lao People’s Democratic Republic

**DOI:** 10.1186/s12889-023-15469-2

**Published:** 2023-03-24

**Authors:** Bounbouly Thanavanh, Nobuyuki Hamajima, Kaiyason Sida, Kene Duangdy, Lasavong Latsamy, Khounsavath Senaphane, Viengsakhone Louangpradith, Souphaphone Sadettan, Souphalak Inthaphatha, Kimihiro Nishino, Eiko Yamamoto

**Affiliations:** 1 Department of Healthcare Administration, Nagoya University Graduate School of Medicine, Nagoya, Japan; 2 Xaiyabouli Provincial Health Office, Lao People’s Democratic Republic, Xaiyabouli, Laos; 3 Xaiyabouli Provincial Hospital, Lao People’s Democratic Republic, Xaiyabouli, Laos; 4 Department of Healthcare and Rehabilitation, Ministry of Health, Lao People’s Democratic Republic, Vientiane, Laos; 5 Cabinet of Ministry of Health, Lao People’s Democratic Republic, Vientiane, Laos

**Keywords:** Lao PDR, Underlying cause of death, Verbal autopsy

## Abstract

**Background:**

In developing countries, it is difficult to collect the data of the underlying cause of death (UCOD), especially when a death does not occur in a health facility. This study aimed to develop a short version of verbal autopsy (VA) and identify the UCOD of adults in Lao People’s Democratic Republic (Lao PDR).

**Methods:**

A short version of VA for deaths outside health facilities was developed. This study included all deaths of people aged 15 years old or older in Xaiyabouli Province in 2020. Socio-demographic factors, place of death, and UCOD of the deceased were collected from health facilities or from family members using a questionnaire including the short VA form. UCOD was compared between home deaths and hospital deaths, between the age group of 15–59 years old and the age group ≥ 60 years old, and between males and females.

**Results:**

Of all the 1,235 deaths included in this study, 1,012 deaths (81.9%) occured at home and 223 deaths (18.1%) at hospitals. The most common UCOD was senility (13.3%), followed by heart/renal failure (10.5%), pneumonia (9.6%) and traffic accident (7.1%). Compared to hospital deaths, home deaths had more people who were females, 75 years old or older, and Lao-Tai. Home deaths had more deaths than hospital deaths due to accident/injury (16.0% vs. 8.1%), tumor (4.7% vs. 1.8%), and senility (16.2% vs. 0%); fewer deaths due to heart/renal disease (15.1% vs. 32.3%), respiratory disease (12.2% vs. 18.8%), liver/gastro-intestine disease (5.3% vs. 9.0%), and infection (3.1% vs. 14.3%). The age group of 15–59 years had more deaths in the categories of accident/injury (28.1% vs. 4.4%), liver/gastro-intestine disease (8.1% vs. 4.4%), infection (7.2% vs. 3.5%), and tumor (6.0% vs. 2.8%). Males had more deaths due to tumor (5.2% vs. 3.0%) and fewer natural deaths (11.2% vs. 15.9%) than females.

**Conclusions:**

The major UCOD category was heart/renal disease in the adult generation in Xaiyabouli Province. Cost-effective interventions based on the multisectoral noncommunicable disease prevention plan should be appropriately implemented. Mortality surveillance using the short VA tool should be conducted for all home deaths in Lao PDR.

**Supplementary Information:**

The online version contains supplementary material available at 10.1186/s12889-023-15469-2.

## Background

Information of deaths, such as the number of deaths, sex and age of the deceased persons, and causes of death, are essential information for policymakers and government officers to develop health policies and to implement appropriate interventions for improving public health in each country [1–3]. In most developed countries, the underlying cause of death (UCOD) is certified by medical doctors and reported to the government using the International Statistical Classification of Diseases and Related Health Problems (ICD), which is the standard medical classification list of the World Health Organization (WHO) [4]. However, many developing countries have no or limited mortality statistics because the systems of vital registration and death certificate by doctors are weak and many people die at home with no access to healthcare services. When a death occurs outside health facilities, the identification of UCOD is more difficult compared to a death at a health facility [4].

A verbal autopsy (VA) is a useful tool to determine the possible cause of death by interviewing family members or care givers regarding signs, symptoms, available medical history, treatment, and circumstances before a death [2, 3, 5]. The WHO started encouraging the use of VA in developing countries and developed reporting forms in the 1970s [2, 6]. The first WHO VA standard tools were developed in 2007, including a questionnaire for the three age groups, cause of death certification, and coding resources consistent with ICD-10, and they were revised in 2014, 2016, and 2022 [1, 3, 5, 7, 8]. There are four methods for VA, namely physician review without algorithmic diagnosis criteria, physician review using an algorithms, computer algorithms, and probabilistic approaches [7], but there is no method with universal advantages because the disease frequency, available personnel, and/or the social and cultural system are different in each country or region [9–11]. However, previous studies suggested that a VA method can provide a rough estimation of UCOD distribution close to that obtained at health facilities if the categories are not so detailed [1, 6].

Lao People’s Democratic Republic (Lao PDR) is a lower-middle income country in Southeast Asia with a population of 7,379,358 in 2021 [12]. It is suggested that deaths due to noncommunicable disease (NCD), such as ischemic heart disease, chronic kidney diseases, diabetes, and chronic respiratory diseases, are increasing in Lao PDR [13–15]. However, previous studies on causes of death in Lao PDR analyzed only deaths at hospitals [16–18]. There have been no government reports on UCOD because the civil registration and death notification systems are weak and lack medical certification. When someone dies at a health facility, healthcare workers issue a death notification that includes the cause of death and a representative of his/her household submits it to the village office and the district home affairs office [19]. When someone dies outside health facilities, his/her family member receives a death notification without the cause of death from the village office and they submit it to the district home affairs office [19, 20]. The information of all deaths is sent from the district home affairs office to the Ministry of Home Affairs. There is a reporting system of the number of hospital deaths from public health facilities to the Ministry of Health, but it includes only maternal deaths and under-five-year-old child deaths. In Lao PDR, approximately 93% of all deaths occur outside of health facilities [20] and 63.9% of all deaths were not registered by civil registration in 2018 [21]. There is a surveillance system of the causes of death but it is also only for maternal deaths and under-five-year-old child deaths at health facilities. Therefore, collecting information of the cause of death in the adult population, especially deaths outside health facilities, is a challenge for the Lao government. The WHO VA instrument for the adult population includes many questions to identify the cause of death [22]. To investigate the causes of death in Lao PDR, especially deaths outside health facilities, a shorter version of the VA instrument is necessary to reduce the investigation time and the budget. Therefore, this study aimed to develop a short version of the VA instrument and identify the causes of death in the adult population in Xaiyabouli Province, Lao PDR.

## Methods

### Development questions to identify UCOD

In this study, a short version of the VA instrument was developed to identify the cause of death of a person who was 15 years or older that occurred outside of health facilities but not maternal death during pregnancy and delivery or within 28 days after childbirth. First, questions for the short VA were listed in the order of clearness in features indicating a category of UCOD; accident/injury, sudden death, stroke (apoplexy), tumor, diarrhea, respiratory disease, tetanus, meningitis, liver failure, heart/renal disease, and senility (natural death). Accident/injury was categorized into suicide/homicide (S/H), bite/sting or food poisoning (B/S), traffic accident (TA), and other injury (OI) (Table 1). Suicide and homicide are recorded in the village office in Lao PDR. Sudden death meant a death within 24 h from onset and classified into myocardial infarction (MI) and arachnoid hemorrhage (AH). When the deceased did not have a chest pain or severe headache, UCOD was other sudden death (OS). When the death occurred within a year after the onset of palsy, UCOD was stroke (ST). When tumors were recognized in the breast, neck, head, abdomen, or other parts in the last month before the death, UCOD was tumor (TU) and the part of the tumor was recorded. When severe diarrhea was found in the last week before the death, UCOD was bloody diarrhea disease (BD) or non-bloody diarrhea disease (ND) according to the characteristics of diarrhea. Respiratory disease was recognized by cough, sputum, and dyspnea and categorized into pneumonia (PN), asthma (AS), and other respiratory disease (OR). Tetanus (TE), meningitis (ME), and liver failure (LF) were decided by typical symptoms and signs. When the deceased had dyspnea when he/she worked, walked, or lied down, UCOD was heart/renal failure (H/R) and other heart disease (OH) according to having edema in the face, legs, ankles, or feet. When the deceased had symptoms other than those included in these questions, the symptoms were recorded. When the deceased had no symptoms or no information from medical records, UCOD was senility (SE) when the age was 70 or older and other disease (OD) when the age was 69 years or younger. UCODs for the short VA instrument were decided considering the situation of Lao PDR and the main causes of deaths in the province from 2016 to 2018.

**Table 1:** Cause of death by short verbal autopsy

Cause of death (code)	Definition	ICD-10	WHO VA 2016 [2]
Suicide/homicide (S/H)	Officially documented (recorded at the village office) suicide/homicide	X71-X83	VA-11.10
Bite/sting/food poisoning (B/S)	Observed or self-reported bite by venomous animals, bite by insects, or poisoned food	A05.9 T62-T63	VA-11.06
Traffic accident (TA)	Documented/observed traffic accident	V00-V99	VA-11.01 VA-11.02
Other injury (OI)	Injury other than the above	S00-T06 T08-T35 T51-T88	VA-11.03–05 VA-11.07–08
Myocardial infarction (MI)	Strong sudden chest pain within 24 h before death	I21	VA-04.02
Arachnoid hemorrhage (AH)	Strong sudden headache within 24 h before death	I60	VA-04.03
Other sudden death (OS)	Sudden death other than the above		
Stroke (ST)	Death within a year after the onset of palsy	I61-I69	VA-04.03
Tumor (TU)	Documented or observed tumors in the last month before the death		
Bloody diarrhea disease (BD)	Severe bloody diarrhea in the last week before the death	A09	VA-01.01
Non-bloody diarrhea disease (ND)	Severe diarrhea in the last week before the death	A04	VA-01.01
Pneumonia (PN)	Cough with sputum, dyspnea and high fever	J09-J18	VA-01.13
Asthma (AS)	Difficulty of breathing (inspiration or expiration) and wheezing	J45	VA-05.02
Other respiratory disease (OR)	Cough, sputum, dyspnea but not pneumonia or asthma		
Tetanus (TE)	Jaw cramping (trismus) 7–10 days after having a wound on the body	A35	VA-01.04
Meningitis (ME)	High fever, stiff neck (discomfort or pain when trying to turn, move, or flex the neck), and vomiting	G00-G03	VA-01.11
Live failure (LF)	Yellow skin, yellow eyes, dark urine, and itchiness in the last week before the death	R17	
Heart/renal failure (H/R)	Dyspnea when exercise or lying down with edema in the face, legs, ankles, or feet	I50,N17-N19	VA-04.05, VA-07.01
Other heart disease (OH)	Dyspnea when exercise or lying down without edema		
Senility (SE)	Death without any symptom and age of 70 years old or older	R41.81	
Other disease (OD)	Death from disease other than the above		

*UCOD* Underlying cause of death, *ICD* International Classification of Disease, *WHO* World Health Organization, *VA* Verbal autopsy

The interview for UCOD started from Question 0 (Q0) “Do you have information on the situation or condition of the deceased?” If an interviewee had no information on the situation or condition of the deceased, the case was excluded from the subjects of the VA. When an interviewee answered yes to Q0, he/she was asked sequentially from Q1 to Q12 (Additional file 1) until UCOD was identified. Q2 was “Did the deceased visit a health facility within a year before the death?” When the answer was yes, the interviewer asked “Were you or a family member explained about the diagnosis possibly being related to the cause of death?” When a death occurred within a year after the last visit to a health facility and the medical record was available, the information from the record was taken into account for determination of UCOD. Except for the homicide/suicide and injury, the categories of UCOD with the frequency intuitively more than 1% were included in the short VA form.

### A short version of the VA instrument

The short VA instrument in this study included four parts: Part 1 was for collecting socio-demographic data of the deceased, namely name, sex, birthday, village name, ethnic group, the educational level, occupation, number of family members, and the date of the death. Part 2 was for collecting the data (name and birthday) of the interviewee, who was a family member of the deceased. Part 3 was a structured questionnaire for assigning the UCOD. Part 4 included the name of the interviewer, the date of interview, and time of interview. The time was measured from Part 1 to the last question of Part 4. The short VA was made to be used by trained healthcare workers. The standard interview time was set to be less than 20 min. The priority was high comparability among different areas in Lao PDR and it was assumed that interviewees would understand the standard Lao language. The draft of the VA short version in Lao language was tested on 19 deaths at Xaiyabouli Provincial Hospital and the final version was made after revision.

### Preliminary verification of the short VA form using hospital deaths

To estimate rough validity of this short VA form, UCOD of people who died at the Xaiyabouli Provincial Hospital between January and September in 2020 were investigated by an interview with a family member using the short VA form and compared to the UCOD in the medical records of the patients. There were 179 deaths at the provincial hospital during the period and 100 deceased patients whose houses were in 19 villages around the hospital were selected. Three nurses of the provincial health department were trained for short VA and all clinical information of the deceased patients were masked to the nurses. The nurse visited the home of the deceased patient 15–30 days after the death and interviewed a family member of the patient who was 18 years old or older. Q2 was excluded from the short VA form to avoid the statement by interviewees on possible UCOD. The completed short VA forms were submitted to the principal researcher to make the final decision to decide the category of the UCOD. Two doctors, other than the principal researcher, reviewed the medical records and decided the UCOD of each patient. The doctors had training about identifying UCOD and UCOD decided by short VA was masked to them. UCODs of 97 deaths (18 females and 79 males) by short VA and in the medical records were compared, because family members of three patients were not at home when the survey team visited. Written informed consent was obtained from a family member of each patient.

### The data of deaths outside medical facilities in Xaiyabouli Province in 2020

The short VA was applied to the deceased people who were 15 years or older and died outside health facilities in Xaiyabouli Province from January to December 2020. The interviewers were 37 nurses and 50 assistant doctors of all district health offices (*n* = 11) and health centers (*n* = 76) in the province. All 87 interviewers attended a training session using the interviewer’s manual. When a death occurs outside health facilities, the death is reported from the head of the village or village health volunteers to the corresponding district health office or health center. The interviewers closely worked with the head of the village or village health volunteers in each village of Xaiyabouli Province. The interviewers visited the household of the deceased 15–30 days after the death. A face-to-face interview was conducted with a family member who was 18 years or older and lived with the deceased in the same household until the day of the death. Informed consent was obtained from the family member before the interview. The time (minutes) of the interview was recorded. In 2020, 1,031 deaths of people aged 15 years or older were reported in the province and 1,012 deaths were investigated using the short VA form because families of 19 deceased people declined to be interviewed. All completed short VA forms were submitted to supervisors to be examined to check that there was no missing information and then submitted to the principal researcher to make the final decision to assign the UCOD.

### The data of deaths at health facilities in Xaiyabouli Province in 2020

In 2020, 223 patients aged 15 years or older died at health facilities in Xaiyabouli Province. The information of the dead patients (hospital deaths) were taken from their medical records including sex, age, ethnic group, occupation, name of the health facility, and cause of death.

### Comparison of categories of UCODs

UCODs were categorized into nine groups, such as accident/injury (suicide/homicide, bite/sting/food poisoning, traffic accident, drowning, and other injury), heart/renal disease (myocardial infarction, heart/renal failure with edema, heart failure, renal failure, other heart disease), respiratory disease (pneumonia, asthma, and other respiratory disease), brain disease (arachnoid hemorrhage and stroke), liver/gastro-intestine disease (liver cirrhosis, liver failure, hepatitis, bloody or non-bloody diarrhea, and gastro-intestine bleeding), infection (tuberculosis, tetanus, meningitis, sepsis, and rickettsia), tumor, senility, and other (unknown and other disease).

All 1,012 deaths outside of health facilities were home deaths and all 223 deaths at health facilities were hospital deaths. The percentages of UCOD categories were compared between home deaths (*n* = 1,012) and hospital deaths (*n* = 223), between the age of 15–59 years old (*n* = 530) and age ≥ 60 years old (*n* = 705), and between males (*n* = 695) and females (*n* = 540).

### Statistical analysis

Data were analyzed using SPSS version 21.0 (IBM SPSS Inc, Amonk, NY, USA). In the verification study, the sensitivity, the specificity, and the positive predictive value of the short VA were calculated based on the hospital diagnosis as a gold standard. Cohen’s kappa was used to estimate the agreement between the short VA and hospital diagnosis and κ > 0.75 was considered excellent agreement [23]. Fisher’s exact test was used for comparing UCOD categories between two groups. A *P*-value < 0.05 was considered statistically significant.

### Ethical issues

This study was approved by the Research Ethical Review Committee of the National Institute of Public Health, Lao PDR (approval number: 049 /NECHR). All methods were performed in accordance with the relevant guidelines and regulations.

## Results

### Development and verification of the short VA form

In this study, the short VA form was developed including 22 UCODs considering the situation of Lao PDR. The UCOD was coded by ICD-10 and corresponding category of WHO VA (Table 1) [2, 24]. The short VA form was validated by comparing UCOD of 97 patients who died at Xaiyabouli Provincial Hospital that were recorded in the medical records and those decided by the short VA. The sensitivity of the short VA was 85.7% (12/14) for myocardial infarction, 80.0% (4/5) for asthma, and 84.6% (11/13) for heart/renal failure with face edema (Table 2). The sensitivity was 90.7% (88/97, 95% confidence interval 83.1–95.7%). The specificity and the positive predictive value of the short VA were 98.8% and 92.3% for myocardial infarction, 97.6% and 84.6% for heart/renal failure with face edema, 98.9% and 88.9% for pneumonia, 98.9% and 87.5% for stroke, respectively. Kappa statistics showed that κ was 0.896, which means a perfect agreement between UCOD by the short VA and hospital diagnoses.

**Table 2:** Comparison of underlying cause of death by short verbal autopsy and diagnosis at hospitals (*N* = 97)

Diagnosis at hospitals (code)	Short verbal autopsy (code)												Total	
	TA	MI	AH	ST	TU	ND	PN	AS	TE	ME	LF	H/R	OD	
Traffic accident (TA)	**12**	0	0	0	0	0	0	0	0	0	0	0	0	12
Myocardial infarction (MI)	0	**12**	0	0	0	0	0	0	0	0	0	1	1	14
Arachnoid hemorrhage (AH)	0	0	**4**	0	0	0	0	0	0	0	0	0	0	4
Stroke (ST)	0	0	0	**7**	0	0	0	0	0	0	0	0	0	7
Tumor (TU)	0	0	0	0	**6**	0	0	0	0	0	0	0	0	6
Non-bloody diarrhea (ND)	0	0	0	0	0	**1**	0	0	0	0	0	0	0	1
Pneumonia (PN)	0	0	0	0	0	0	**8**	0	0	0	0	0	0	8
Asthma (AS)	0	0	0	0	0	0	1	**4**	0	0	0	0	0	5
Tetanus (TE)	0	0	0	0	0	0	0	0	**1**	0	0	0	0	1
Meningitis (ME)	0	0	0	0	0	0	0	0	0	**1**	0	0	0	1
Liver failure (LF)	0	0	0	0	0	0	0	0	0	0	**2**	0	0	2
Heart/renal failure (H/R)	0	1	0	1	0	0	0	0	0	0	0	**11**	0	13
Other (OD)	0	0	3	0	0	0	0	0	0	0	0	1	**19**	23
Total	12	13	7	8	6	1	9	4	1	1	2	13	20	**97**

### Characteristics of deaths in Xaiyabouli Province in 2020

**Fig. 1:**
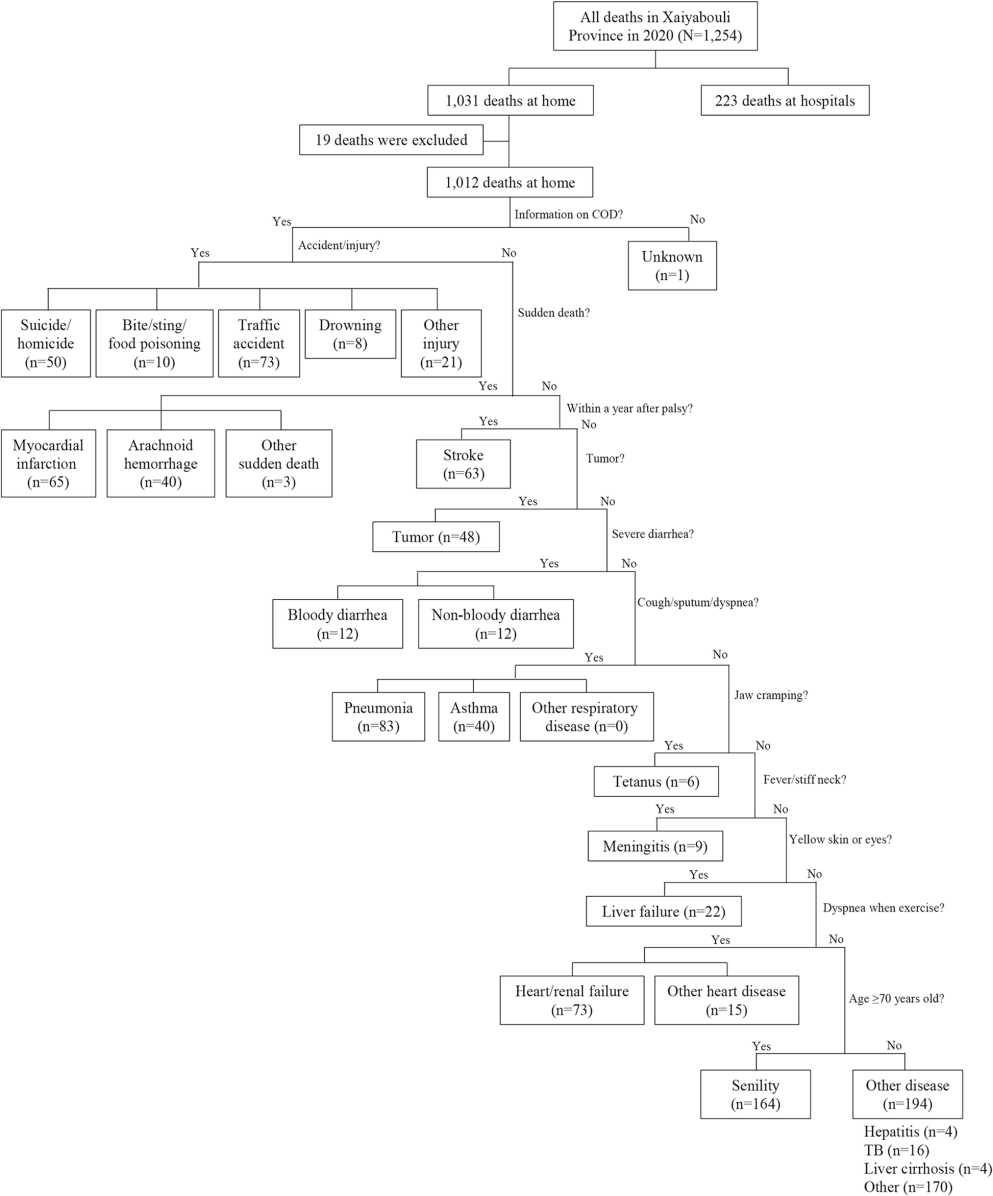
Flowchart of underlying causes of death by verbal autopsy. There were 1,254 deaths in Xaiyabouli Province in 2020, including 1,031 home deaths and 223 hospital deaths. Underlying causes of 1,012 home deaths were identified using the short version of the verbal autopsy form developed in this study.

In 2020, a total of 1,254 deaths in Xaiyabouli Province were reported and the information of 1,235 deaths were collected including 1,012 deaths outside health facilities and 223 deaths at health facilities. All 1,012 deaths outside health facilities were at home (81.9%) and all 223 deaths at hospitals (18.1%) but not health centers (Table 3). The information of 1,012 home deaths was collected using the short VA form (Fig. 1).

**Table 3:** Characteristics of deaths in Xaiyabouli Province in 2020 (*N* = 1,235)

Characteristics	Total (*N* = 1,235)	Home death (*N* = 1,012)	Hospital death (*N* = 223)	P
	N (%)	n (%)	n (%)	
Sex				0.044
Male	695 (56.3)	556 (54.9)	139 (62.3)	
Female	540 (43.7)	456 (45.1)	84 (37.7)	
Age group (years old)				< 0.001
15–29	105 (8.5)	76 (7.5)	29 (13.0)	
30–44	160 (13.0)	118 (11.7)	42 (18.8)	
45–59	265 (21.5)	207 (20.5)	58 (26.0)	
60–74	352 (28.5)	289 (28.6)	63 (28.3)	
75 ≤	353 (28.6)	322 (31.8)	31 (13.9)	
Ethnic group				< 0.001
Lao-Tai	1,021 (82.7)	858 (84.8)	163 (73.0)	
Mon-Khmer	136 (11.0)	106 (10.5)	30 (13.5)	
Hmong-Mien	52 (4.2)	22 (2.2)	30 (13.5)	
Others	26 (2.1)	26 (2.6)	0 (0.0)	
Education				-
None	-	316 (31.2)	-	
Primary	-	441 (43.6)	-	
Secondary	-	148 (14.6)	-	
High-school	-	80 (7.9)	-	
University or higher	-	27 (2.7)	-	
Occupation				< 0.001
Farmer	957 (77.5)	763 (75.4)	194 (87.0)	
Employee	214 (17.3)	185 (18.3)	29 (13.0)	
Other	64 (5.2)	64 (3.6)	0 (0.0)	
Place of death				-
Provincial Hp	170 (13.8)	0 (0.0)	170 (76.2)	
District Hp	53 (4.3)	0 (0.0)	53 (23.8)	
Home	1,012 (81.9)	1,012 (100.0)	0 (0.0)	
Interviewee				-
Wife	-	275 (27.2)	-	
Husband	-	195 (19.3)	-	
Father	-	26 (2.6)	-	
Mother	-	21 (2.1)	-	
Daughter	-	409 (40.4)	-	
Son	-	70 (6.9)	-	
Other	-	16 (1.6)	-	
Interview time				-
< 10 min	-	242 (23.9)	-	
10–19 min	-	770 (76.1)	-

Of all the 1,235 deaths in 2020, most deaths were males (*n* = 695, 56.3%), 60–74 years old (*n* = 352, 28.5%) and 75 years or older (*n* = 353, 28.6%), Lao-Tai ethnic group (*n* = 1,021, 82.7%), and farmers (*n* = 957, 77.5%) (Table 3). Compared to hospital deaths, home deaths had more women (45.1% vs. 37.7%, *P* = 0.044), the age group ≥ 75 years old (31.8% vs. 13.9%), and Lao-Tai ethnic group (84.8% vs 73.0%). In terms of occupation, farmers were more likely to die at hospitals (87.0%) compared to at home (75.4%). Most interviewees of short VA were daughters (*n* = 409, 40.4%), wives (*n* = 275, 27.2%), and husbands (*n* = 195, 19.3%). The average interview time was 12.4 min (range, 3–19 min) and most interviews were 10–19 min (*n* = 770, 76.1%).

### UCOD of all deaths in Xaiyabouli Province

UCOD of the 1,235 deaths were identified using the short VA or from medical records at hospitals (Fig. 1). The most common cause was senility (*n* = 164, 13.3%), followed by heart/renal failure (*n* = 130, 10.5%), pneumonia (*n* = 119, 9.6%), traffic accident (*n* = 88, 7.1%), myocardial infarction (*n* = 79, 6.4%), and stroke (*n* = 78, 6.3%) (Table 4). Heart failure or renal failure could not be separately identified as UCOD of home deaths (*n* = 73, 7.2%), although heart failure (*n* = 26) and renal failure (*n* = 31) were diagnosed for hospital deaths. Of all deaths, 50 deaths (4.0%) were due to suicide/homicide and 52 deaths (4.2%) due to tumors, and causes of 183 deaths (14.8%) were categorized into others because UCOD was not identified. The major UCOD category was heart/renal disease (*n* = 225, 18.2%) followed by accident/injury (*n* = 180, 14.6%), respiratory disease (*n* = 165, 13.4%), senile (*n* = 164, 13.3%), and brain disease (*n* = 129, 10.4%).

**Table 4:** Comparison of underlying cause of death according to place of death, age group, and sex

Underlying cause of death	Total (*N* = 1,235)	Place of death			Age group (years old)			Sex		
		Home (*N* = 1,012)	Hospital (*N* = 223)	P	15–59 (*N* = 530)	60 ≤ (*N* = 705)	P	Male (*N* = 695)	Female (*N* = 540)	P
	N (%)	N (%)	N (%)		N (%)	N (%)		N (%)	N (%)	
**Accident/injury**	**180 (14.6)**	**162 (16.0)**	**18 (8.1)**	**0.002**	**149 (28.1)**	**31 (4.4)**	** < 0.001**	**102 (14.7)**	**78 (14.4)**	**0.935**
Suicide/homicide	50 (4.0)	50 (4.9)	0 (0.0)		33 (6.2)	17 (2.4)		26 (3.7)	24 (4.4)	
Bite/sting/food poisoning	13 (1.1)	10 (1.0)	3 (1.3)		11 (2.1)	2 (0.3)		8 (1.2)	5 (0.9)	
Traffic accident	88 (7.1)	73 (7.2)	15 (6.7)		81 (15.3)	7 (1.0)		53 (7.6)	35 (6.5)	
Drowning	8 (0.6)	8 (0.8)	0 (0.0)		8 (1.5)	0 (0.0)		5 (0.7)	3 (0.6)	
Other injury	21 (1.7)	21 (2.1)	0 (0.0)		16 (3.0)	5 (0.7)		10 (1.4)	11 (2.0)	
**Heart/renal disease**	**225 (18.2)**	**153 (15.1)**	**72 (32.3)**	**< 0.001**	**97 (18.3)**	**128 (18.2)**	**1.000**	**134 (19.3)**	**91 (16.9)**	**0.298**
Myocardial infarction	79 (6.4)	65 (6.4)	14 (6.3)		37 (7.0)	42 (6.0)		45 (6.5)	34 (6.3)	
Heart/renal failure	130 (10.5)	73 (7.2)	57 (25.6)		55 (10.4)	75 (10.6)		76 (10.9)	54 (10.0)	
Other heart disease	16 (1.3)	15 (1.5)	1 (0.4)		5 (0.9)	11 (1.6)		13 (1.9)	3 (0.6)	
**Respiratory disease**	**165 (13.4)**	**123 (12.2)**	**42 (18.8)**	**0.012**	**57 (10.8)**	**108 (15.3)**	**0.022**	**95 (13.7)**	**70 (13.0)**	**0.737**
Pneumonia	119 (9.6)	83 (8.2)	36 (16.1)		46 (8.7)	73 (10.4)		68 (9.8)	51 (9.4)	
Asthma	40 (3.2)	40 (4.0)	0 (0.0)		9 (1.7)	31 (4.4)		23 (3.3)	17 (3.1)	
Other respiratory disease	6 (0.5)	0 (0.0)	6 (2.7)		2 (0.4)	4 (0.6)		4 (0.6)	2 (0.4)	
**Brain disease**	**129 (10.4)**	**103 (10.2)**	**26 (11.7)**	**0.545**	**56 (10.6)**	**73 (10.4)**	**0.925**	**70 (10.1)**	**59 (10.9)**	**0.640**
Arachnoid hemorrhage	51 (4.1)	40 (4.0)	11 (4.9)		28 (5.3)	23 (3.3)		28 (4.0)	23 (4.3)	
Stroke	78 (6.3)	63 (6.2)	15 (6.7)		28 (5.3)	50 (7.1)		42 (6.0)	36 (6.7)	
**Liver/gastro-intestine disease**	**74 (6.0)**	**54 (5.3)**	**20 (9.0)**	**0.043**	**43 (8.1)**	**31 (4.4)**	**0.008**	**39 (5.6)**	**35 (6.5)**	**0.547**
Liver cirrhosis	10 (0.8)	4 (0.4)	6 (2.7)		3 (0.6)	7 (1.0)		5 (0.7)	5 (0.9)	
Liver failure	25 (2.0)	22 (2.2)	3 (1.3)		21 (4.0)	4 (0.6)		17 (2.4)	8 (1.5)	
Hepatitis	4 (0.3)	4 (0.4)	0 (0.0)		3 (0.6)	1 (0.1)		4 (0.6)	0 (0.0)	
Bloody diarrhea diseases	12 (1.0)	12 (1.2)	0 (0.0)		5 (0.9)	7 (1.0)		4 (0.6)	8 (1.5)	
Non-bloody diarrhea diseases	13 (1.1)	12 (1.2)	1 (0.4)		7 (1.3)	6 (0.9)		5 (0.7)	8 (1.5)	
Gastro-intestine bleeding	10 (0.8)	0 (0.0)	10 (4.5)		4 (0.8)	6 (0.9)		4 (0.6)	6 (1.1)	
**Infection**	**63 (5.1)**	**31 (3.1)**	**32 (14.3)**	**< 0.001**	**38 (7.2)**	**25 (3.5)**	**0.006**	**37 (5.3)**	**26 (4.8)**	**0.795**
Tuberculosis	16 (1.3)	16 (1.6)	0 (0.0)		7 (1.3)	9 (1.3)		9 (1.3)	7 (1.3)	
Tetanus	7 (0.6)	6 (0.6)	1 (0.4)		5 (0.9)	2 (0.3)		4 (0.6)	3 (0.6)	
Meningitis	10 (0.8)	9 (0.9)	1 (0.4)		8 (1.5)	2 (0.3)		7 (1.0)	3 (0.6)	
Sepsis	29 (2.3)	0 (0.0)	29 (13.0)		17 (3.2)	12 (1.7)		17 (2.4)	12 (2.2)	
Rickettsia	1 (0.1)	0 (0.0)	1 (0.4)		1 (0.2)	0 (0.0)		0 (0.0)	1 (0.2)	
**Tumor**	**52 (4.2)**	**48 (4.7)**	**4 (1.8)**	**< 0.001**	**32 (6.0)**	**20 (2.8)**	**< 0.001**	**36 (5.2)**	**16 (3.0)**	**0.018**
Cervical cancer	4 (0.3)	4 (0.4)	0 (0.0)		3 (0.6)	1 (0.1)		0 (0.0)	4 (0.7)	
Breast cancer	2 (0.2)	2 (0.2)	0 (0.0)		1 (0.2)	1 (0.1)		0 (0.0)	2 (0.4)	
Breast abscess	1 (0.1)	1 (0.1)	0 (0.0)		1 (0.2)	0 (0.0)		0 (0.0)	1 (0.2)	
Liver cancer	4 (0.3)	4 (0.4)	0 (0.0)		2 (0.4)	2 (0.3)		4 (0.6)	0 (0.0)	
Lung cancer	4 (0.3)	4 (0.4)	0 (0.0)		3 (0.6)	1 (0.1)		4 (0.6)	0 (0.0)	
Renal cancer	2 (0.2)	2 (0.2)	0 (0.0)		0 (0.0)	2 (0.3)		2 (0.3)	0 (0.0)	
Spinal cancer	2 (0.2)	2 (0.2)	0 (0.0)		0 (0.0)	2 (0.3)		2 (0.3)	0 (0.0)	
Nodular melanoma	1 (0.1)	1 (0.1)	0 (0.0)		1 (0.2)	0 (0.0)		1 (0.1)	0 (0.0)	
Abdominal tumor	27 (2.2)	24 (2.4)	3 (1.3)		17 (3.2)	10 (1.4)		19 (2.7)	8 (1.5)	
Neck tumor	4 (0.3)	4 (0.4)	0 (0.0)		3 (0.6)	1 (0.1)		4 (0.6)	0 (0.0)	
Colon cancer	1 (0.1)	0 (0.0)	1 (0.4)		1 (0.2)	0 (0.0)		0 (0.0)	1 (0.2)	
**Senility**	**164 (13.3)**	**164 (16.2)**	**0 (0.0)**	**< 0.001**	**0 (0.0)**	**164 (23.3)**	**< 0.001**	**78 (11.2)**	**86 (15.9)**	**0.018**
**Other**	**183 (14.8)**	**174 (17.2)**	**9 (4.0)**	**< 0.001**	**58 (10.9)**	**125 (17.7)**	**0.001**	**104 (15.0)**	**79 (14.6)**	**0.936**

The percentage represents the proportion of the deaths due to the cause to a total number of deaths in each group

### Comparison of UCOD between home deaths and hospital deaths, between age groups, and between males and females

When the UCOD categories were compared between home deaths and hospital deaths, home deaths had more accident/injury (16.0% vs. 8.1%, *P* = 0.002) and tumor (4.7% vs. 1.8%, *P* < 0.001) (Table 4). All deaths due to senility were home deaths. Deaths caused by heart/renal disease (15.1% vs. 32.3%, *P* < 0.001), respiratory disease (12.2% vs. 18.8%, *P* = 0.012), liver/gastro-intestine disease (5.3% vs. 9.0%, *P* = 0.043), and infection (3.1% vs. 14.3%, *P* < 0.001) were less likely to have occurred at home compared to at hospitals.

All deaths were divided into the age group of 15–59 years old and the age group of 60 years old or older. The age group of 15–59 years had more deaths of the categories of accident/injury (28.1% vs. 4.4%, *P* < 0.001), liver/gastro-intestine disease (8.1% vs. 4.4%, *P* = 0.008), infection (7.2% vs. 3.5%, *P* = 0.006), and tumor (6.0% vs. 2.8%, *P* < 0.001) (Table 4). Comparison of UCOD between males and females showed that males had significantly fewer natural deaths (11.2% vs. 15.9%, *P* = 0.018) and more deaths due to tumor (5.2% vs. 3.0%, *P* = 0.018) than females.

## Discussion

In this study, the short VA was developed to identify UCODs of deaths outside health facilities in Xaiyabouli Province. The preliminary validation of the short VA form by hospital diagnosis showed high sensitivity and specificity. However, UCOD of 17.2% of home deaths could not be identified by the short VA and heart failure and renal failure could not be distinguished. Symptoms of chronic heart failure and end-stage renal failure are similar and the two diseases are major progressive factors of each other [25]. To identify more kinds of UCOD, being diagnosed and having laboratory examinations at health facilities before deaths is needed. The WHO VA instrument includes questions to ask about the medical history, results of laboratory exams, and diagnosis by healthcare professional [22]. However, access to healthcare service is poor in rural areas and provinces and basic laboratory tests for high-income countries, such as biochemical tests and pathological tests, are not regularly performed or unavailable even at provincial hospitals [17, 26–28]. Therefore, the short VA form was useful to understand the causes of most home deaths by interviewing family members for a short time but the form should be improved based on a more accurate larger-size validation study.

To the best of our knowledge, this is the first study to show the UCODs in the adult population in Lao PDR, including deaths outside hospitals. In this study, heart/renal disease was the major UCOD category among all deaths as well as hospital deaths and one of the main categories among home deaths. Deaths due to arachnoid hemorrhage and stroke accounted for 10.4% of all deaths and 69 deaths categorized into other UCOD had a history of hypertension or diabetes mellites. The results of this study suggest that 46.2% of all deaths were due to NCD (heart/renal disease, respiratory disease, brain disease, and tumor), which was lower than that estimated by the WHO in 2016 [29]. In the WHO estimation, 60% of all deaths were due to NCD, including cardiovascular disease (27%), cancer (12%), chronic respiratory disease (5%), diabetes (4%), and others (12%). In 2014, the Lao government implemented a multisectoral action plan to prevent NCD by reducing risks (tobacco use, harmful alcohol consumption, unhealthy diet, and physical inactivity) and promoting treatments (cardiovascular disease, diabetes mellitus, and cancer) [30]. Not only the implementation of cost-effective interventions but also the monitoring of the morbidity and mortality of NCD are essential to help reduce NCD in the whole country.

Characteristics of home deaths were different from hospital deaths. Home deaths had more deceased who were females and 75 years old or older. It may be because all natural deaths were home deaths and the life expectancy of women (70 years old) is longer than that of men (66 years old) [31, 32]. The result that the Lao-Tai ethnic group had more home deaths suggests that beliefs are different among ethnic groups. It is reported that people of Lao ethnic groups (Lao Loum, Lao Theung, and Lao Sung) believe that the spirits of people are not reincarnated but become malevolent ones when the people die by accident or during childbirth [33]. The educational level cannot be compared between home deaths and hospital deaths. When the educational levels of people who died at home were compared with the average educational levels in the general population, home deaths in this study had more people who had no education (31.2% vs. 16.0%) and fewer people who had education at university or higher (2.7% vs. 14.3%) [34]. People with a higher educational level may have treatment at health facilities more than those with a lower educational level.

In this study, the percentages of UCOD categories were different between home deaths and hospital deaths. Home deaths had more deaths due to accident/injury, tumor, and senility than hospital deaths. On the other hand, hospital deaths had more deaths due to acute illness, such as heart or renal failure, pneumonia, gastro-intestine bleeding, and sepsis. In Lao PDR, most people focus on acute illness but not on disease prevention or health promotion and people often come to health facilities after diseases are advanced. Lao people prefer to die at home because they believe that the soul of a person may wander and not be reincarnated when he or she dies outside the home [33]. Therefore, when patients are diagnosed as having uncurable diseases, they prefer going to traditional healers rather than visiting health facilities for treatment. When a patient at a hospital is going to die, their family hopes that the patient will return to their home before dying. Senility was the major cause of home deaths and all deaths due to senility were at home. If an elderly person who died at home due to senility visited a health facility, they might be diagnosed with heart disease, cerebral disease, or pneumonia as the cause of death [35, 36].

Comparing the UCODs between males and females, deaths due to senility were significantly higher among females than males. This is because of longer life expectancy among females than males. The average life-year of females was also older (62.6 years) than that of males (59.7 years) in this study. Deaths due to tumor, especially abdominal tumor, were more likely to occur in males compared to females. In a previous study on cancer mortality at hospitals in 2007–2008, the major cancer for death was liver cancer among both males and females, but of all cancer deaths the percentage of liver cancer death was higher among males (52.2%) than females (28.4%) [18]. Another study including hospital deaths in Vientiane Capital in 2013–2015 showed that liver cancer accounted for 1.9% of all deaths among males, which was higher than among females (0.8%) [16]. According to the WHO data on mortality in Lao PDR, the major cancer among males was liver cancer but that among females was breast cancer [37]. Abdominal tumor in this study may suggest ascites due to cancer, especially liver cancer. Liver cancer may be the major cancer in Lao PDR due to the high prevalence of HBV and HCV and poor access to health service for receiving appropriate treatment [38, 39].

Compared to the age group of 60 years or older, the age group of younger than 60 years had higher mortality due to accident/injury, liver/gastro-intestine disease, infection, and tumor, especially traffic accident, suicide/homicide, and liver failure. These results were consistent with those of previous studies in Lao PDR and other developing countries [15, 16, 40–42]. In this study, all deaths due to drowning occurred in the younger age group. It may be because Xaiyabouli Province is mountainous and has a hydroelectric dam using the Mekong River. It is reported that most people with liver disease die aged between 18 to 65 years [43] and that mortality due to cancer in the young generation is higher in low- and middle-income countries compared to high-income countries [27]. To reduce preventable deaths in the younger generation, interventions for preventing traffic accidents, promoting mental health service, and establishing a safe environment for people are needed in the province.

This study has some limitations. First, UCOD of 17.2% of home deaths could not be identified using the short VA form. Heart failure and renal failure could not be distinguished and causes of diarrhea or liver failure could not be found. Second, this study may not include all deaths that occurred in the province because 63.9% of all deaths in the whole country were not registered using civil registration in 2018 [21]. Third, the results of this study cannot be representative of the data of Lao PDR because this study included only deaths in a province for a year. The data of both hospital deaths and home deaths should be collected in all provinces and analyzed to identify the causes of death of the adult population. To collect UCOD of home deaths, the adult mortality surveillance by healthcare workers using a short VA tool should be routine work for district health offices and health centers [23, 44].

## Conclusions

This study showed that the major UCOD category was heart/renal disease in the adult generation of Xaiyabouli Province in 2020 and that 81.9% of all deaths were home deaths. Compared to hospital deaths, home deaths had more deceased who were women, who were ≥ 75 years old, and who were Lao-Tai ethnic group. Home deaths had more deaths due to accident/injury, tumor, and senility but fewer deaths due to heart/renal disease, respiratory disease, liver/gastro-intestine disease, and infection. The age group of 15–59 years had more deaths of the categories of accident/injury, liver/gastro-intestine disease, infection, and tumor. Males had more deaths due to tumor and fewer natural deaths than females. Cost-effective interventions based on the multisectoral NCD prevention plan should be appropriately implemented. To understand UCOD of all deaths in the country, mortality surveillance using the short VA tool should be conducted for all home deaths, although the short VA needs to be revised based on a large country-wide validation study.

## Supplementary Information

**Additional file 1.** Short verbal autopsy form for adult deaths outside health facilities in Lao PDR.

## Data Availability

The datasets analyzed in this study are not publicly available due to institutional policies but are available from the corresponding author on reasonable request.

## Abbreviations

ICD: International Statistical Classification of Diseases and Related Health Problems
Lao PDR: Lao People’s Democratic Republic
NCD: Noncommunicable disease
UCOD: Underlying cause of death
VA: Verbal autopsy
WHO: World Health Organization
