# Supplementary material for: Assessment of the underlying causes of adult deaths using a short version of verbal autopsy in Xaiyabouli Province, Lao People’s Democratic Republic
Source: BMC Public Health. 2023 Mar 24;23:560. doi: 10.1186/s12889-023-15469-2 (PMC10037893; doi:10.1186/s12889-023-15469-2)
Supplement: Supplementary file 1 — Additional file 1. Short verbal autopsy form for adult deaths outside health facilities in Lao PDR. [file 12889_2023_15469_MOESM1_ESM.doc]

Additional file 1 Short verbal autopsy form for adult deaths outside health facilities in Lao PDR

|  | Question | Response | Cause of death | Code |
| --- | --- | --- | --- | --- |
| Q0 | Do you have information on the situation or condition of the deceased? | Yes | (To Q1) |  |
|  |  | Not at all | Unknown (stop) | UN |
| Q1 | Accident/injury or disease? | Accident/injury | (To Q1-1) |  |
|  |  | Disease | (To Q2) |  |
| Q1-1 | Suicide/homicide? | Yes | Suicide/homicide (stop) | S/H |
|  |  | No | (To Q1-2) |  |
| Q1-2 | Bite by venomous animals, bite by insects, or eat poisoned food in the last 30 days before the death? | Yes | Bite/sting/food poisoning (stop) | B/S |
|  |  | No | (To Q1-3) |  |
| Q1-3 | Serious injury due to a traffic accident in the last 30 days before the death? | Yes | Traffic accident (stop) | TA |
|  |  | No | Other injury (stop) | OI |
| Q2 | Did the deceased visit a medical facility within one year before the death? | Yes | Suggested UCOD: ……………… (To Q3) |  |
|  |  | No | (To Q3) |  |
| Q3 | Sudden death within 24 hours from the onset of some symptoms? | Yes | (To Q3-1) |  |
|  |  | No | (To Q4) |  |
| Q3-1 | With chest pain? | Yes | Myocardial infarction (stop) | MI |
|  |  | No | (To Q3-2) |  |
| Q3-2 | With severe headache? | Yes | Arachnoid hemorrhage (stop) | AH |
|  |  | No | Other sudden death with symptoms: ............  (stop) | OS |
| Q4 | Death within a year after the onset of palsy? | Yes | Stroke (stop) | ST |
|  |  | No | (To Q5) |  |
| Q5 | Tumors in the breast, neck, head, abdomen, or others in the last month before the death? | Yes | (To Q5-1) |  |
|  |  | No | (To Q6) |  |
| Q5-1 | Where was the tumor? |  | Tumor…………(stop) | TU |
| Q6 | Severe diarrhea in the last week before the death? | Yes | (To Q6-1) |  |
|  |  | No | (To Q7) |  |
| Q6-1 | Severe bloody diarrhea? | Yes | Bloody diarrhea disease (stop) | BD |
|  |  | No | Non-bloody diarrhea disease (stop) | ND |
| Q7 | Cough/sputum/dyspnea? | Yes | (To Q7-1) |  |
|  |  | No | (To Q8) |  |
| Q7-1 | With a fever higher than 38 ℃? | Yes | Pneumonia (stop) | PN |
|  |  | No | (To Q7-2) |  |
| Q7-2 | Before the death, did he/she have difficulty of breathing (inspiration or expiration) and wheezing? | Yes | Asthma (stop) | AS |
|  |  | No | Other respiratory disease (stop) | OR |
| Q8 | Jaw cramping (trismus) 7-10 days after having a wound on the body? | Yes | Tetanus (stop) | TE |
|  |  | No | (To Q9) |  |
| Q9 | High fever, stiff neck (discomfort or pain when trying to turn, move, or flex the neck) and vomiting? | Yes | Meningitis (stop) | ME |
|  |  | No | (To Q10) |  |
| Q10 | Yellow skin, yellow eyes, dark urine, and itchiness in the last week before the death? | Yes | Liver failure (stop) | LF |
|  |  | No | (To Q11) |  |
| Q11 | Shortness of breath (dyspnea) when exercise or lying down? | Yes | (To Q11-1) |  |
|  |  | No | (To Q12) |  |
| Q11-1 | With edema in the face/legs/ankles/feet? | Yes | Heart/renal failure with edema (stop) | H/R |
|  |  | No | Other heart diseases (stop) | OH |
| Q12 | With other symptoms? | Yes | Symptom names: ………………. (stop) |  |
|  |  | No | (To Q12-1) |  |
| Q12-1 | Aged 70 years or older | Yes | Senility (stop) | SE |
|  |  | No | Other disease (stop) | OD |

UCOD, underlying cause of death.
